# Highest Defoliation Tolerance in *Amaranthus cruentus* Plants at Panicle Development Is Associated With Sugar Starvation Responses

**DOI:** 10.3389/fpls.2021.658977

**Published:** 2021-06-07

**Authors:** Ismael Cisneros-Hernández, Erandi Vargas-Ortiz, Estefany S. Sánchez-Martínez, Norma Martínez-Gallardo, Daniela Soto González, John Paul Délano-Frier

**Affiliations:** ^1^Centro de Investigación y de Estudios Avanzados del IPN, Unidad Irapuato, Irapuato, Mexico; ^2^Facultad de Agrobiología, Universidad Michoacana de San Nicolás de Hidalgo, Uruapan, Mexico; ^3^Universidad Iberoamericana, Plantel León, León, Mexico

**Keywords:** grain amaranth, carbon re-allocation, defoliation, development, master regulators of metabolism, invertases, sugar starvation response, trehalose-6-phosphate

## Abstract

Defoliation tolerance (DT) in *Amaranthus cruentus* is known to reach its apex at the panicle emergence (PE) phase and to decline to minimal levels at flowering (FL). In this study, defoliation-induced changes were recorded in the content of non-structural carbohydrates and raffinose family oligosaccharides (RFOs), and in the expression and/or activity of sugar starvation response-associated genes in plants defoliated at different vegetative and reproductive stages. This strategy identified sugar-starvation-related factors that explained the opposite DT observed at these key developmental stages. Peak DT at PE was associated with increased cytosolic invertase (CI) activity in all organs and with the extensive induction of various class II trehalose-phosphate synthase (*TPS*) genes. Contrariwise, least DT at FL coincided with a sharp depletion of starch reserves and with sucrose (Suc) accumulation, in leaves and stems, the latter of which was consistent with very low levels of CI and vacuolar invertase activities that were not further modified by defoliation. Increased Suc suggested growth-inhibiting conditions associated with altered cytosolic Suc-to-hexose ratios in plants defoliated at FL. Augmented cell wall invertase activity in leaves and roots, probably acting in a regulatory rather than hydrolytic role, was also associated with minimal DT observed at FL. The widespread contrast in gene expression patterns in panicles also matched the opposite DT observed at PE and FL. These results reinforce the concept that a localized sugar starvation response caused by C partitioning is crucial for DT in grain amaranth.

## Introduction

The ability of plants to cope with leaf-tissue loss caused by herbivory or mechanical damage is a plastic trait influenced by environmental conditions (Maschinski and Whitham, 1989). Some factors affecting plant responses to defoliation by herbivory are the timing of herbivore attack (Feeny, 1970), its intensity (Del-Val and Crawley, 2005), the resources available to the plant facing this stress (Mattson, 1980; Juenger and Bergelson, 1997), and plant age (Maschinski and Whitham, 1989; Del-Val and Crawley, 2005). Because of the differences in resource allocation and architecture through plant ontogeny, the ability to cope with herbivory/mechanical damage-related defoliation is likely to change as plants develop (Boege and Marquis, 2005; Boege et al., 2007). Less studied factors influencing compensatory ability and tolerance in plants are the sowing time and seasonal changes in light and temperature (Malone et al., 2002).

Some of the physiological mechanisms associated with damage compensation in tolerant plants include pre-existing high levels of carbon (C) storage in roots, the plant’s ability to reallocate these C reserves to other tissues, and the activation of dormant meristems (Strauss and Agrawal, 1999). For example, rapid utilization of C reserves was shown to sustain leaf regrowth in *Lolium perenne* in the first hours after compensation of defoliation and also after repeated grazing (Morvan-Bertrand et al., 2001; Lee et al., 2010). Such response was associated with an increment of sucrose (Suc) transport and the *de novo* synthesis of Suc transporters (Berthier et al., 2009). Likewise, the accumulation of below-ground C reserves in certain shrubs allowed full compensation to defoliation in terms of fruit output (Rivera-Solís et al., 2012), whereas other plants rely on the preferential resource allocation to vegetative tissues for regrowth, at the cost of fruit production (Fang et al., 2008). In addition, meristem activation is known to confer an improved ability to compensate for damage in plants having an indeterminate growth habit (Rosenthal and Welter, 1995). Although the above mechanisms can partially explain compensation and the expression of tolerance, there is limited information about the physiological, biochemical, and molecular processes that support them.

Grain amaranths are an under-exploited non-graminaceous crop that produce seeds similar to cereals, hence their denomination as pseudo-cereals (Berghofer and Schoenlechner, 2002). Grain amaranths are native of America and comprise three species: *Amaranthus cruentus*, *A. hypochondriacus*, and *A. caudatus* (Shukla et al., 2003). *A cruentus* is a grain amaranth of neutral photoperiod that can produce two crops per year in certain latitudes (Espitia-Rangel et al., 2010). This and other amaranth species have been shown to be tolerant to defoliation. They also can compensate and even over-compensate in response to a large fraction of foliar tissue loss (i.e., 50–100%) and/or repeated defoliation events (Marquis, 1996; Martínez-Moreno et al., 1999; Castrillón-Arbeláez et al., 2012; Vargas-Ortiz et al., 2013; Hoidal et al., 2019, 2020). In *A. cruentus*, this capacity was related with rapid carbohydrate reallocation from root and stems after defoliation, which was accompanied by changes in sucrolytic and amylolytic gene expression and activity in these organs (Castrillón-Arbeláez et al., 2012; Vargas-Ortiz et al., 2013). A subsequent study found that severe DT in grain amaranth was highly dependent on the development stage of the plant, since almost complete recovery of biomass and grain yield was maintained if defoliation occurred prior to flowering stage, at which defoliation tolerance became highly compromised. Tolerance to defoliation was also found to be affected by the season in which leaf loss occurred, being higher in the spring–summer. This capacity was associated with levels of phosphoenolpyruvate carboxylase (PEPC) activity in leaves (Vargas-Ortiz et al., 2015).

However, there is little information about how tolerance capacity is maintained during different stages of plant development. In this study, the hypothesis proposing that C reserve accumulation and utilization vary during *A. cruentus* ontogeny and that this variation constitutes a critical factor affecting the plant’s ability to resume growth and reproduce after severe defoliation was tested. It was further stated that severe defoliation leads to a temporary energy deprivation response akin to that produced by extended darkness or exposure to (a) biotic stress conditions (Arias et al., 2014; Henry et al., 2014; Lin et al., 2014). This energy deprivation response is coordinated by known regulators of metabolism, such as SUCROSE-NON-FERMENTING-1α-RELATED KINASE (SnRK1α) and Target of Rapamycin (TOR) complex protein kinases, which, together with trehalose-6-phosphate (Tre6P), reprogram plant metabolism to optimize the use of available energy to ensure survival and reproduction (Liu et al., 2013; Yu et al., 2015; Figueroa and Lunn, 2016).

In the present study, grain amaranth plants were completely defoliated and several parameters were determined in four major organs (i.e., roots, stems, leaves, and panicles) at four different developmental stages [i.e., early and late vegetative stages, panicle emergence (PE) and flowering (FL)]. These were compared to those determined in undamaged plants. The first parameters measured included steady-state accumulation and enzyme activities characteristic of starch reserve and soluble non-structural carbohydrates (NSCs) pools. The second set of parameters analyzed was the gene expression of enzymes from the Tre6P and trehalose (Tre) metabolism, and the third was that of regulators of metabolism and SnRK_2_ kinases. The analysis was extended to include raffinose-family oligosaccharides (RFOs), RFO biosynthesis-related enzyme genes, and PEPC. This experimental design was justified by the fact that soluble NSCs and RFOs can accumulate in many plants in response to different stress conditions, where they play roles as osmoprotectants, ROS scavengers, or protein stabilizers. RFOs originate from the binding of myo-inositol (MI) and UDP-galactose to generate galactinol (Gol) that subsequently contributes to the formation of raffinose (Raf), stachyose (Sta), verbascose (Ver), and possibly higher-order RFOs, by acting as a galactose unit donor (Peterbauer and Richter, 2001; Sengupta et al., 2015). RFOs can also function in C storage and redistribution (Ayre et al., 2003; Nishizawa et al., 2008; ElSayed et al., 2014). Similarly, starch can be rapidly mobilized to provide soluble sugars in response to salt drought and other stresses, including defoliation (Castrillón-Arbeláez et al., 2012; Vargas-Ortiz et al., 2013; Reguera et al., 2013; González-Rodríguez et al., 2019) in order to maintain cell turgor or protect membranes and proteins from stress-related damage. Moreover, Suc metabolism, via invertases, can provide hexoses having the potential to regulate abiotic stresses responses and plant development (Ruan, 2012; Barnes and Anderson, 2018; Pignocchi et al., 2021). Analysis of genes coding for proteins involved in the biosynthesis and degradation of Tre and for proteins conforming the SnRK1α and TOR complexes was justified by their role in the preservation of energy homeostasis *via* the reprogramming of plant metabolism in response to stress-related resource deprivation (Baena-González and Hanson, 2017). In addition, the analysis of genes belonging to the plant-exclusive SnRK2 subgroup was considered on the basis of their capacity to regulate stress-related metabolic responses associated with sugar limitation (Halford and Hey, 2009; Lou et al., 2017; Vishwakarma et al., 2017).

The utilization of the above experimental strategy permitted the identification of several sugar starvation-related factors that were associated with the highly contrasting defoliation tolerance of *A. cruentus* plants observed at the PE and FL developmental stages. Their contribution to defoliation tolerance is discussed in the context of the efficient management of reduced energy levels to ensure plant survival during stress.

## Materials and Methods

### Plant Material and Experimental Design

Plant samples analyzed in this study were obtained from the experimental procedure described by Vargas-Ortiz et al. (2015). Briefly, 20-day-old grain amaranth plants (*A. cruentus* cv. Tarasca) grown in 18-L pots containing a general soil mixture were placed under greenhouse conditions during the spring–summer of 2014. Next, plants were subjected to severe defoliation after reaching two different vegetative and reproductive development stages, respectively. The vegetative stages were defined as “vegetative 1” (V1) and “vegetative 2” (V2), respectively. Plants at V1 were utilized 4–5 weeks after germination (wag) and had 6–8 expanded leaves, whereas V2 plants were used at 6–7 wag and had 11–14 expanded leaves. The two reproductive stages were defined as panicle emergence (PE) and flowering (FL), respectively. Plants at PE were defoliated 9–10 wag, while those at FL were damaged 11–12 wag, when they had reached ∼50% anthesis. Defoliation was done with a punch-hole borer. The defoliation treatment was completed in 3 days, at a rate of 1/3 daily loss of total foliar in the first 2 days. On the third day, the leaf tissue remaining after a 66% percent leaf area loss was cut from the base of the petiole to reach total plant defoliation and was immediately placed in liquid nitrogen for subsequent analysis. These represent the “Leaf” component used in all further experimentation. Twenty-four hours later, leaves, roots, stems, and panicles (when applicable) were sampled from intact plants (controls), whereas only roots, stems, and panicles were collected from defoliated plants. All plant organs were flash frozen in liquid nitrogen for subsequent analysis.

### Quantitation of Non-structural Carbohydrates (NSCs) and Raffinose Family Oligosaccharides (RFOs)

For soluble NSCs, 20 mg of lyophilized ground tissue was extracted three times with 0.6 ml of extraction buffer (50 mM Hepes KOH, pH 7.4, and 5 mM MgCl_2_ in 80% ethanol) at 700 rpm and 80°C for 15 min. The extracts were centrifuged at 2,900 × g for 10 min and the resulting supernatants were combined. Suc, glucose (Glc), and fructose (Fru) were determined enzymatically in the supernatants using a coupled assay with glucose-6-phosphate dehydrogenase (from yeast, grade II, Roche, Mannheim Germany) in which NADPH formation was measured at 340 nm (Zhao et al., 2010). For starch, the pellets resulting from the above procedure were re-suspended in 0.5 ml of 10 mM KOH and autoclaved for 30 min. Starch was hydrolyzed overnight at 37°C in 0.7 ml of starch degradation buffer [100 mM Hepes, pH 7.5, 3 mM MgCl_2_, 10 U of amylase (α-amylase), Type VI-B, from porcine pancreas (E.C. number 3.2.1.1; Sigma-Aldrich Chemical, St. Louis MO, United States) and 10 U of amyloglucosidase from *Aspergillus niger* (E.C. number 3.2.1.3; Sigma)]. After centrifugation at 2,900 × g, the supernatants were stored at 4°C. The pellet was re-extracted with 0.5 ml of starch degradation buffer at 37°C for 30 min. After centrifugation, the supernatants were combined and assayed enzymatically for Glc as described above.

Identification and determination of RFOs content in leaf, stem, and root samples were performed by High-Performance Anion-Exchange Chromatography with Pulsed Amperometric Detection (HPAEC–PAD), as described previously (Mellado-Mojica et al., 2016). All chemicals used to standardize the chromatographic separation and for RFOs quantitation were from Sigma (Sigma-Aldrich Chemical). RFOs quantified were MI, Gol, Raf, Sta, and Ver.

### Enzymatic Assays: Invertases, Suc Synthase, and Amylase

The extraction procedures to determine invertase (EC 3.2.1.26) and Suc synthase (Susy, EC 2.4.1.13) activities were done as described previously (Wright et al., 1998), using 25 mg of lyophilized ground plant tissue as the starting material. Samples were re-suspended in 0.5 ml of extraction buffer [50 mM Hepes KOH, pH 8, 5 mM MgCl_2_, 2 mM EDTA, 1 mM MnCl_2_, 1 mM CaCl_2_, 1 mM dithiothreitol (DTT), and 1 mM of the phenylmethylsulfonyl fluoride (PMSF) proteinase inhibitor]. Vacuolar (VI), cytosolic (CI) invertase, and Susy activities were quantified in these extracts after being clarified by centrifugation. The remaining pellets were washed three times with extraction buffer and were used to determine cell wall invertase activity (CWI). For invertase assays, the reaction mixtures contained 50 μl of the above extracts, 100 μl of 0.5 M Suc, and 100 μl of reaction buffer (citrate-phosphate buffer pH 5.0, 5.5, or 7.0 for CWI, VI, or CI, respectively) at 37°C for 30 min. Free Glc was measured as described above. Susy activity levels were determined as described by Wright et al. (1998).

Total amylase was quantified as described by Vargas-Ortiz et al. (2013) using 20 mg of ground lyophilized plant tissue as starting material. Extraction was done with 500 μl of extraction buffer (50 mM MOPS KOH, pH 7.5, 20 mM MgCl_2_, 2 mM CaCl_2_, 1 mM EDTA, 1 mM DTT). Starch degradation was measured by the colorimetric detection of the reducing sugars released using 3, 5-dinitrosalicylic acid.

### Phosphoenol Pyruvate Carboxylase Assay (PEPC)

PEPC was assayed as described previously (Vargas-Ortiz et al., 2015). Briefly, 20 mg of lyophilized ground leaf tissue was extracted with 0.5 ml of PEPC extraction buffer. For maximal activity, 2.5 mM PEP and 10 mM NaHCO_3_ were used in the assay. Reaction was started with 2-μl aliquots of the PEPC extracts.

### Quantitative RT-PCR

The quantitative gene expression analysis was performed as described previously (González-Rodríguez et al., 2019). Briefly, total RNA was extracted from frozen root, stem, leaf, and panicle tissues using the Trizol reagent (Invitrogen, Carlsbad, CA, United States) according to Palmeros-Suárez et al. (2015). Quantitative gene expression analysis, performed using SYBR Green detection chemistry (Bio-Rad, Hercules, CA, United States), was determined following the procedure detailed by these workers. Primer design for the amplification of the amaranth gene transcripts included in this study was facilitated by the use of the following bio-informatic tools: Primer3^1^, Beacon Designer^2^, UNAFold^3^, and Oligo Evaluator^4^. Primer sequence was based on recently published genomic data (Clouse et al., 2016) and was validated using reported methods (Thornton and Basu, 2011). Relative gene expression data were obtained using the comparative cycle threshold method (Livak and Schmittgen, 2001) using two grain amaranth housekeeping genes for data normalization: *AhACT7* and *AhEF1a*. Most genes analyzed in this study were extensively described before (González-Rodríguez et al., 2019).

The analysis included the following genes: (i) the class I *TREHALOSE PHOSPHATE SYNTHASE* (*TPS*) *AhTPS1* [*At-Bv*] gene, involved in the synthesis of trehalose-6-phospahte (Tre6P), the first step in the Tre biosynthetic pathway (Lunn et al., 2014); (ii) all grain amaranth class II TPS genes (i.e., *AhTPS5* [*At-Bv*], *AhTPS6* [*At*]; *AhTPS7* [*At-Bv*], *AhTPS8* [*At*], *AhTPS9* [*At-Bv*], *AhTPS10* [*At*], and *AhTPS11* [*Bv*]); (iii) three *TREHALOSE PHOSPHATE PHOSPHATASE* (*TPP*) genes (i.e., *AhTPPA* [*At-Bv*], *AhTPPD* [*At-Bv*], and *AhTPPI* [*At-Bv*]); (iv) the only *TREHALASE* (TRE) gene identified in the *A. hypochondriacus* genome (i.e., *AhTRE;* [*At-Bv*]); (v) components of the regulators of metabolism *SnRK1*α (*AhSnRK1*α*-KIN10* [*At-Bv*]) and *SnRK1*α ACTIVATING KINASE (*AhSnRAK* [*GRIK1*; *At*]); (vi) three orthologs of *Arabidopsis thaliana SnRK2* genes, two of which, *AhSnRK2.6* and *AhSnRK2.7*, resemble those that positively regulate ABA signaling in *Arabidopsis* (Sah et al., 2016), and genes involved in the synthesis of RFOs, *AhGolS1* [*At-Bv*], *AhGolS*2 [*At-Bv*], *AhRafS* [*At*], and *AhStaS* [*At*]. This study also included the analysis of several invertase and Suc synthase genes: two cell wall invertase genes (*AhCWI1* [*At*] and *AhCWI3* [*At*]), three vacuolar invertase genes (*AhVI2* [*Bv*], *AhVI4* [*At*], and *AhVI3* [*At*]), five cytosolic invertases (*AhCIA* [*At*], *AhCI2-1* [*Bv*], *AhCI1* [*Bv*], *AhCI2-*2 [*At-Bv*], and *AhCIB* [*At*]), and three Suc synthase genes (*AhSUS3* [*At*], *AhSUS1* [*Bv*], and *AhSUS4* [*At*]). Additionally, the *AhSnRK2.3* [*At*] gene and genes coding for the three protein components of the plant TOR complex, *AhTOR* [*At-Bv*], *AhRAPTOR* [*At-Bv*], and *AhLST8* [*At-Bv*], were analyzed as well. The primers used for the amplification of the latter genes are listed in Supplementary Table 1. All genes are annotated in the *A. hypochondriacus* genome (Clouse et al., 2016), and most were named according to the closest homology shown with their respective *A. thaliana* (*At*) and/or *Beta vulgaris* (*Bv*) orthologs (Cisneros-Hernández, 2016; González-Rodríguez et al., 2019).

### Statistical Analysis

The study had a factorial design. “Defoliation” was the first factor with two levels: undamaged plants (“undefoliated controls”) and total defoliation (“defoliated plants”). “Development stage” was the second factor, in which four levels were established: two vegetative (V1 and V2) and two reproductive (PE and FL) stages, respectively. The “plant organ” was the third factor analyzed, with four levels: roots, stems, leaves, and panicles. Nine plants divided into three-plant pools were destined for each intersection of the first two factors. All variables were analyzed with ANOVAs, including treatment, development stage, and their interactions as sources of variation. These analyses were followed by Tukey HSD tests (*P* ≤ 0.05) using the R package (R Core Team, 2020, version 3.2.0)^5^.

## Results

This study’s objective was to identify sugar starvation/carbon mobilization responses that could be associated with either enhancing or depressing defoliation tolerance in *A. cruentus*, during the PE and FL development stages, respectively. Fluctuations in levels of NSCs and RFOs, sucrolytic and amylolytic enzyme activity, and their respective genes were analyzed. Changes in the expression levels of Tre biosynthesis and hydrolysis genes, in addition to genes coding for proteins associated with master regulators of metabolism, were also analyzed.

### Changes in NSCs Levels, Sucrolytic and Amylolytic Activity, and Expression of Related Genes

**FIGURE 1:**
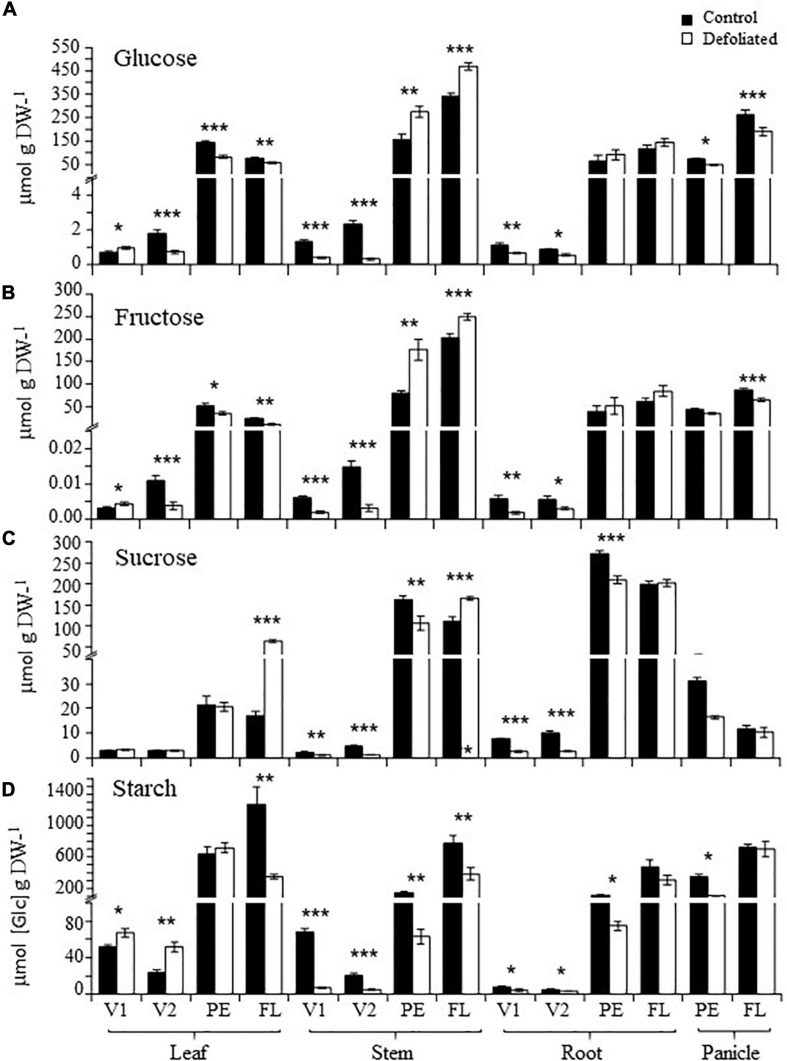
Non-structural carbohydrate content in organs of defoliated *versus* non-defoliated *Amaranthus cruentus* plants. Levels of (**A**) glucose, (**B**) fructose, (**C**) sucrose, and (**D**) starch were determined in leaves, stems, roots, and panicles of *A. cruentus* plants subjected to complete defoliation at four different development stages: Vegetative 1 (V1), vegetative 2 (V2), panicle emergence (PE), and flowering (FL). The bars represent the means ± standard error of three technical replicates obtained from three pooled plant samples produced by combining organs from nine individual plants. The results are from a representative experiment that was replicated twice. Asterisks indicate statistically significant differences between treatments at *P < 0.05, **P < 0.01, or ***P < 0.001 for a one-way ANOVA, Tukey HSD test.

**FIGURE 2:**
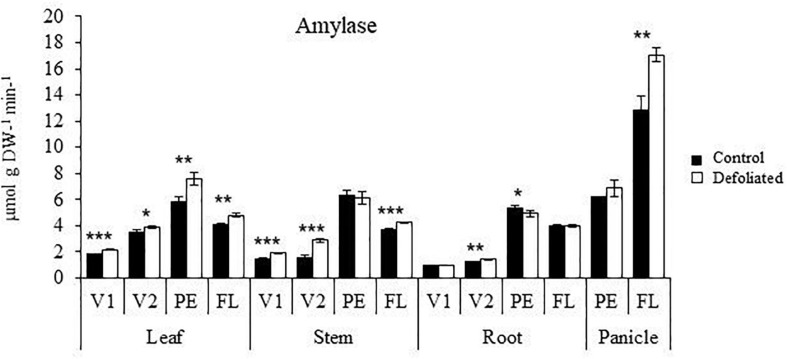
Amylase activity in organs of defoliated *versus* non-defoliated *Amaranthus cruentus* plants. Amylase activity was quantified in leaves, stems, roots, and panicles of *A. cruentus* plants subjected to complete defoliation at four different development stages: Vegetative 1 (V1), vegetative 2 (V2), panicle emergence (PE), and flowering (FL). The bars represent the means ± standard error of three technical replicates obtained from three pooled plant samples produced by combining organs from nine individual plants. The results are from a representative experiment that was replicated twice. Asterisks indicate statistically significant differences between treatments at *P < 0.05, **P < 0.01, or ***P < 0.001 for a one-way ANOVA, Tukey HSD test.

The basal levels of soluble and insoluble NSCs increased sharply in all vegetative organs examined during the transition from the vegetative to reproductive stages of development in *A. cruentus* plants (Figure 1). Within this context, the opposite defoliation tolerance detected at PE and FL was reflected by the differential ways in which the developmentally augmented Suc and starch reserves were subsequently affected by this stress in all organs examined. While Suc levels were either reduced or remained unaffected by defoliation, at PE, they significantly increased in stems and leaves of defoliated *A. cruentus* plants at FL and remained unchanged in roots and were reduced in panicles (Figure 1C). Inversely to Suc, the high basal starch levels recorded in leaves, stems, and roots during FL underwent a significant reduction in response to defoliation while they remained mostly unchanged or were reduced less drastically in equivalent organs, at PE (Figure 1D). This pattern was reversed in panicles, where starch depletion in response to defoliation was more pronounced at PE (Figure 1D). Reduced starch content in defoliated *A. cruentus* plants, at FL, coincided with the observed induction of amylase activity in leaves, stems, and panicles (Figure 2).

**FIGURE 3:**
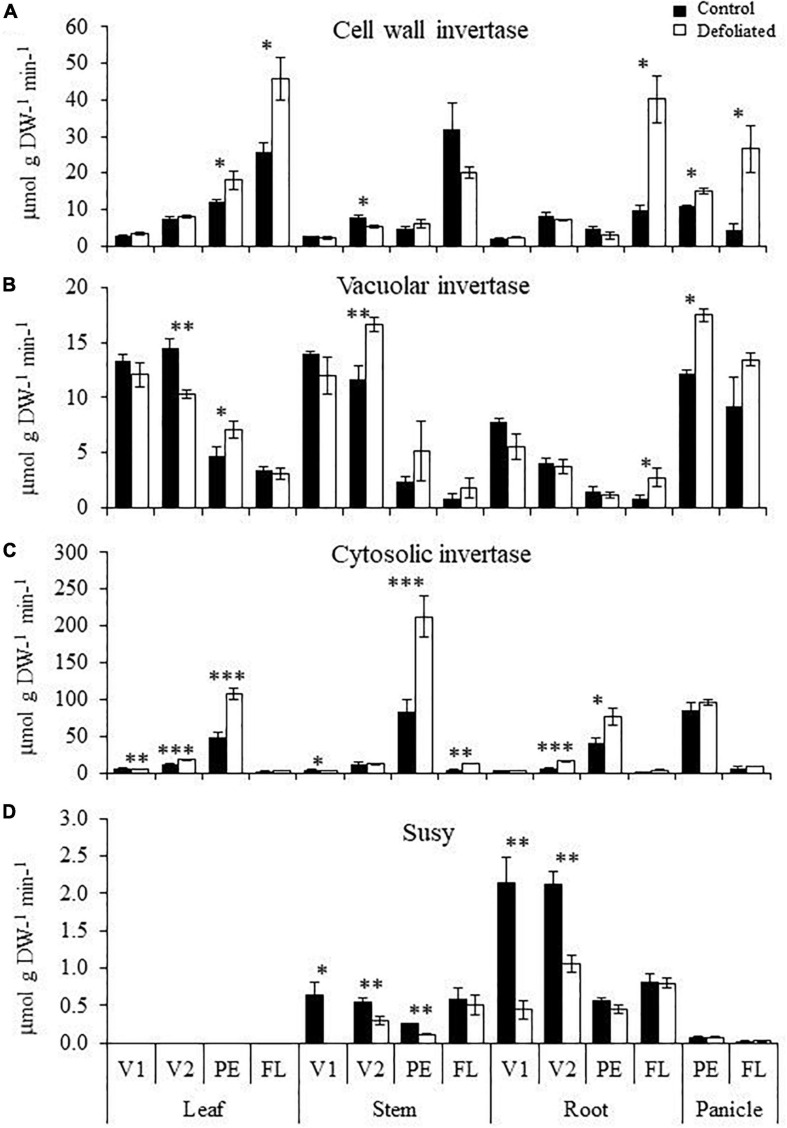
Invertase and sucrose synthase activity in organs of defoliated *versus* non-defoliated *Amaranthus cruentus* plants. Levels of **(A)** cell wall, **(B)** vacuolar, **(C)** cytosolic invertase, and **(D)** sucrose synthase (SuSy) activities were measured in leaves, stems, roots, and panicles of *A. cruentus* plants subjected to complete defoliation at four different development stages: Vegetative 1 (V1), vegetative 2 (V2), panicle emergence (PE), and flowering (FL). The bars represent the means ± standard error of three technical replicates obtained from three pooled plant samples produced by combining organs from nine individual plants. The results are from a representative experiment that was replicated twice. Asterisks indicate statistically significant differences between treatments at ^∗^*P* < 0.05, ^∗∗^*P* < 0.01, or ^∗∗∗^*P* < 0.001 for a one-way ANOVA, Tukey HSD test.

The highest basal CI activity was recorded at PE, in all organs examined. A further and significant increase of CI activity was produced in leaves, stems, and roots of defoliated *A. cruentus* plants at this stage (Figure 3C). This was contrary to basal CI and VI activity at FL, which was generally the lowest recorded in all organs and was not further affected by defoliation, except for the repression of CI activity in stems (Figures 3B,C). Conversely, the highest basal CWI activity levels were mostly detected at FL. These were significantly increased by defoliation in leaves and roots (Figure 3A). A significant induction of CWI and VI activity in leaves, at PE, was also registered (Figures 3A,B). Defoliation-induced changes of invertase activity in the panicle included activation of CWI, at both PE and FL, and of VI, only at PE. No changes in CI activity were detected (Figure 3). No SuSy activity was detected in leaves while it remained unchanged or was significantly reduced in response to defoliation in all other plant organs analyzed (Figure 3D).

The changes detected in invertase activity were consistent with reduced/unchanged Suc levels observed in organs of defoliated *A. cruentus* during PE, but not FL. A coincidence was also found between the significantly higher Glc and Fru levels and induced CI activity in stems of defoliated plants, at PE (Figures 1A,B, 3C).

Increased defoliation-induced CWI activity levels coincided with the induced expression of *AhCW1*, in leaves, at PE, and of *AhCW3*, in roots, at FL, while augmented VI activity in leaves and panicles of defoliated plants, at PE, coincided with the induction of *AhVI2* and *4* and *AhVI2*, respectively (Supplementary Figure 1). Conversely, the gene expression data of the *AhCI* genes analyzed did not coincide with the high basal CI activity, further increased by defoliation, detected in all vegetative organs examined, at PE. Likewise, transcript abundance of the three *AhSuSy* genes examined did not coincide with the Susy activities recorded.

### Modified Expression of Trehalose Biosynthesis and Degradation Genes

**FIGURE 4:**
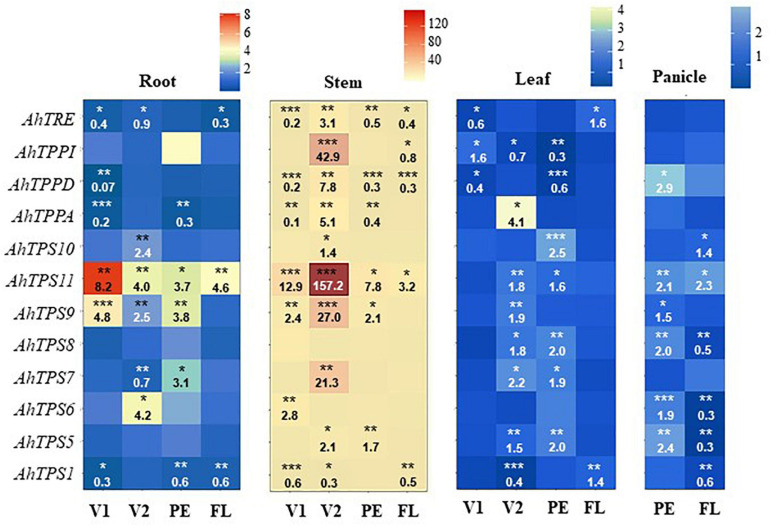
Changes in the expression of trehalose metabolism genes in defoliated *versus* non-defoliated *Amaranthus cruentus* plants. Data shown compare completely defoliated versus intact *A. cruentus* plants at four different developmental stages: vegetative 1 (V1), vegetative 2 (V2), panicle emergence (PE), and flowering (FL). RNA was extracted for each stage from the following tissues: roots, stems, leaves, and panicles. qRT-PCR was then performed for genes coding for enzymes involved in trehalose-6-phosphate (Tre6) synthesis (trehalose phosphate synthase, TPS class I: *AhTPS1*), signaling (TPS, class II: *AhTPS5-11*), Tre6P de-phosphorylation (trehalose phosphate phosphatase, TPP: *AhTPPA*, *D* and *I*), and trehalose degradation (trehalase, TRE, *AhTRE*). Relative expression was quantified based on the 2^–ΔΔ*CT*^ calculation (Livak and Schmittgen, 2001). C_T_ values for all genes were normalized to the C_T_ value of the *AhACT7* and *AhEF1a* housekeeping genes. Then, the changes in gene expression were calculated from four technical replicates. Asterisks indicate statistically significant differences with undefoliated controls: *P < 0.05, **P < 0.01, or ***P < 0.001 (*n* = 4) for a one-way ANOVA, Tukey HSD test. Data shown are representative of an experiment that was replicated twice. Changes are visualized using a different color code for each tissue, as indicated in the upper section of the figure.

In general, the expression of the *AhTPS1* gene remained unchanged or was repressed in response to defoliation in all vegetative organs and developmental stages examined. The only exception observed was its induction in leaves, at FL (Figure 4). *AhTPS5* was not induced in roots. Its induction in stems, leaves, and panicles, at PE, contrasted with the general insensitivity/repression of this gene to defoliation, at FL. Similar contrasts in expression patterns, at PE and FL, were produced by the *AhTPS7*, *AhTPS8*, and *AhTPS9* genes. A limited defoliation-induced expression of *AhTPS6* was only detected in the V1 and V2 stages; the only exception was the induction of this gene in panicles, at PE. The induction of *AhTPS10* in response to defoliation was restricted only to certain organs and developmental stages. Apart from its exclusive induction in roots and stems, at V2, *AhTPS10* was only activated in leaves, at PE, and in panicles, at FL. With the exception of leaves, where *AhTPS11* was not induced by defoliation, at FL, this gene was broadly induced by defoliation in all other plant organs analyzed irrespective of the development stage examined.

Apart from the induction of *AhTPPD* in panicles, at PE, the expression of the three *AhTPP* genes tested remained unaltered or was repressed by defoliation, at PE and FL. Similarly, a widespread neutral or repressed expression of *AhTRE* was detected, except for the exclusive induction of this gene in leaves of defoliated plants, at FL (Figure 4). The highly contrasting differences in the expression pattern of all *AhTPS* genes observed in the panicle at PE (mostly induced) and FL (mostly repressed or unchanged) suggested that their behavior in this organ might also contribute to the opposite DT observed in these two developmental stages.

### Modified Gene Expression of Stress-Related Regulators of Metabolism

The expression pattern of genes coding for proteins related to the TOR and for *AhSnRK1*α metabolic regulators had no association with opposite DT observed at PE and FL, respectively (Supplementary Figure 2). A widespread defoliation-induced expression of *SnRK2.7* was observed in stems and roots, but without obvious differences between PE and FL.

### Modified Levels of Raffinose Family Oligosaccharides (RFO), RFO Precursors, and Expression of RFO Biosynthetic Genes

Except for the defoliation-induced up-regulation of *AhGolS1* in leaves and panicles, at FL, and of *AhRafS* in roots, at V1, analysis of all genes associated with the synthesis of RFOs showed that they were either repressed or remained unchanged by defoliation. The singular induction of *AhGolS1* in these plant organs, at FL, could have contributed to the minimal DT observed at this development stage (Supplementary Figure 3).

**FIGURE 5:**
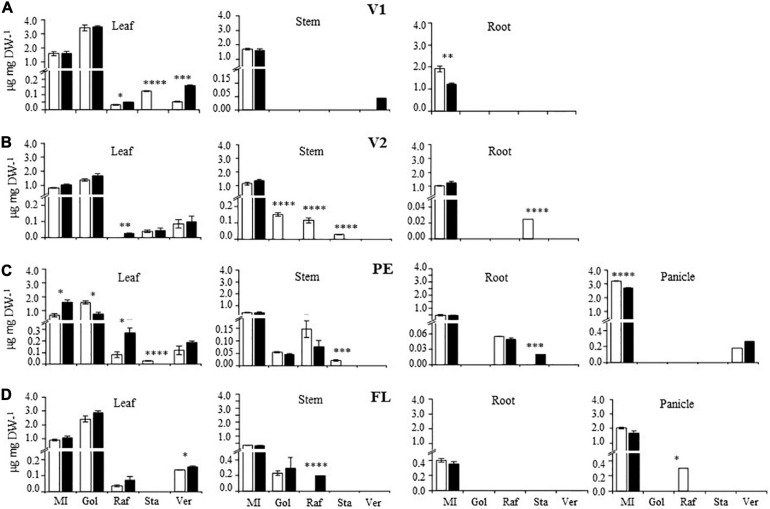
Content of raffinose family oligosaccharides (RFOs) in organs of defoliated *versus* non-defoliated *Amaranthus cruentus* plants. Levels of the RFO precursors, myo-inositol (MI), and galactinol (Gol), and of raffinose (Raf), stachyose (Sta), and verbascose (Ver) RFOs were determined in leaves, stems, roots, and panicles of *A. cruentus* plants subjected to complete defoliation at four different development stages: **(A)** vegetative 1 (V1), **(B)** vegetative 2 (V2), **(C)** panicle emergence (PE), and **(D)** flowering. The bars represent the means ± standard error of three technical replicates obtained from three pooled plant samples produced by combining organs from nine individual plants. The results are from a representative experiment that was replicated twice. Asterisks indicate statistically significant differences between treatments at ^∗^*P* < 0.05, ^∗∗^*P* < 0.01, ^∗∗∗^*P* < 0.001, or ^****^P < 0.0001 for a one-way ANOVA, Tukey HSD test.

The dominant defoliation-related downregulation of RFO biosynthetic genes that suggested low RFO-biosynthetic activity was generally in accordance with the prominent accumulation of the MI and Gol RFO precursors in all development stages examined (Figure 5). Basal MI content tended to decrease in all vegetative organs as plants developed, mainly in stems and roots, but increased again in panicles, whereas basal Gol accumulated mostly in leaves, but smaller amounts also gradually accumulated in stems of older plants. Only at PE were the already high content levels of foliar MI increased, and those of Gol decreased, by defoliation. Defoliation-induced accumulation of Raf was also observed in leaves, at PE, and in stems, at FL. A slight accumulation of Sta was also observed in roots of defoliated plants, at PE. Modified RFO accumulation patterns in panicles were again in accordance with the opposite defoliation tolerance observed at PE and FL. Thus, MI content was reduced by defoliation at PE, but not FL, while the basal Raf content, detected in panicles only at FL, was lowered in response to defoliation.

### Changes in PEPC Activity

Basal PEPC activity in leaves was drastically reduced as plants developed and was not significantly affected by defoliation (Supplementary Figure 4). Relatively stable, but lower, basal PEPC activity was detected in stems. The activity detected in stems was similar to that measured in leaves at PE and FL and was mostly repressed by defoliation, except at V2.

## Discussion

In common with most other plant stress conditions, defoliation in grain amaranth has been observed to negatively affect its energy reserve status (Castrillón-Arbeláez et al., 2012; Vargas-Ortiz et al., 2013). Yet, contrary to other crops, defoliation may boost productivity if the stress is partial and performed at certain development stages (Martínez-Moreno et al., 1999; Vargas-Ortiz et al., 2015; Hoidal et al., 2019, 2020). Further evidence showed, however, that tolerance to severe mechanical defoliation in grain amaranth drastically drops at FL after reaching its apex at PE (Vargas-Ortiz et al., 2015). The increased defoliation tolerance observed in *A. cruentus* plants as they developed from their early (V1) and late vegetative (V2) stages to the PE reproductive stage was previously associated to high foliar PEPC activity at V1 and V2 stages, which was interpreted as a key factor leading to the gradual accumulation of starch and Suc reserves in stems and roots that later sustained optimal recovery after leaf loss at PE (Vargas-Ortiz et al., 2015). In the present study, this developmentally defined PEPC activity pattern was not affected by defoliation. Curiously, the low PEPC basal activity levels detected in stems, although extensively reduced by defoliation, appeared not to have influenced the opposite DT observed at PE and FL.

The present study also revealed that Suc and starch were differentially impacted by defoliation at PE and FL. In accordance to Vargas-Ortiz et al. (2015), the levels of these two NSCs were higher in these two reproductive developmental stages than in the preceding vegetative stages. This pattern agreed with the high demand for energy, C, and nutrients that need to be exported to the shoot apical meristem during the transition to flowering. In perennial and other plants, C demands can be supplied, in part, by the mobilization of stored carbohydrate reserves, mostly starch (Chapin et al., 1990; Corbesier et al., 1998, 2002; Castrillón-Arbeláez et al., 2012; Ida et al., 2012; Vargas-Ortiz et al., 2013, 2015). However, the differential changes in Suc and starch that were detected in response to defoliation in an organ-dependent way offered the first clue toward the possible mechanisms responsible for the opposite defoliation tolerance observed at PE and FL. Thus, the high Suc levels present in these two reproductive stages were generally reduced by defoliation, at PE, but increased, at FL. The negative effects on DT at FL caused by augmented Suc levels in stems and leaves were reminiscent of the marked developmental and physiological defects observed in *A. thaliana* cytosolic invertase mutant plants having elevated Suc levels. These alterations were interpreted as a consequence of both low carbon availability for metabolism and growth and a disrupted sugar signaling resulting from altered cytosolic Suc-to-hexose ratios (Pignocchi et al., 2021). In accordance with this study, basal CI activity at FL was lowest in leaves, roots, and panicles of amaranth plants and was not further affected by defoliation. The sharp contrast with the distinctively high CI activity levels observed at PE supported a role for CI and other invertases as regulators of DT in grain amaranth (see below). This possibility is sustained by their proposed central participation in carbon mobilization in *A. thaliana* and other plant species (Welham et al., 2009; Yao et al., 2009; Barnes and Anderson, 2018; Pignocchi et al., 2021). Increased Suc levels may have also contributed to the sharp decrease in starch reserves observed in several organs of defoliated *A. cruentus* plants, at FL, similarly to the above-mentioned study in which levels of starch, as well as glycolytic intermediates, were significantly reduced in roots of cytosolic invertase mutant plants having abnormally high Suc contents due to restricted Suc hydrolytic capacity (Pignocchi et al., 2021).

Moreover, total amylase activity increased with plant age and was higher in defoliated plants. Thus, the induced amylase activity in leaves, stems, and panicles of defoliated amaranth plants, at FL, was also in agreement with the drastic reduction of their starch reserves. This behavior confirmed previous observations made in severely defoliated grain amaranth where an active mobilization of C reserves, mostly starch, coincided with the induced activity of various sucrolytic and amylolytic enzymes (Castrillón-Arbeláez et al., 2012; Vargas-Ortiz et al., 2013).

As previously noted, modified invertase activity/gene expression in response to defoliation were additional factors potentially associated with determining defoliation tolerance amplitude in *A. cruentus*. The gradual increase to maximum levels of CWI activity in amaranth plants as they reached FL, which was further augmented in response to defoliation, was not accompanied by significant reduction in Suc levels. However, this increase was in accordance with the relationship that these sucrolytic enzymes have with reproductive processes, critically so in seed development (Patrick, 1997; Weber et al., 2005). It also agreed with data suggesting that CWIs play a key role in ovule initiation in *A. thaliana* by impacting the sugar signaling network via the regulation of hexose transporters, receptor-like kinases, auxin signaling, and MADS-box transcription factors, rather than by influencing C partitioning through its sucrolytic activity (Liao et al., 2020).

Another mechanism that could explain the lack of coincidence between *CWI* and *CI* gene expression and invertase activity, at PE and FL, is the possible post-translational regulation of these sucrolytic enzymes via endogenous invertase inhibitor proteins. Invertase inhibitors have been reported in several plant species including grain amaranths (Castrillón-Arbeláez et al., 2012). Even though the physiological role of invertase inhibitors remains uncertain, it has gradually emerged that post-translational modulation of invertase impacts numerous metabolic pathways, cellular and physiological processes, as well as molecular regulatory networks, including those controlling development and pathogen defense responses (Bonfig et al., 2010; Yan et al., 2019; Su et al., 2020).

Moreover, high CWI activity at FL contrasted with the lowest VI activity levels recorded during development. In addition, VI activity, at FL, was mostly unaffected by defoliation. Thus, low VI responsiveness to defoliation could have further contributed to the poor defoliation tolerance of *A. cruentus*, at FL, considering the relationship that VI activity has with the maintenance of active and accelerated growth (Ruan et al., 2010; Ruan, 2014). Likewise, CI could be related to the setting of the reproductive stage in grain amaranth, as shown by the CI activity peak reached, at PE, in all organs analyzed. In other plant models, CIs have been related to reproduction (Jia et al., 2008) and other high energy-demanding processes (Martín et al., 2013). In addition, induced CI activity in defoliated plants at PE probably contributed to the enhanced defoliation tolerance observed by maintaining sink strength under low sugar conditions, while raised CWI, VI, and CI activities in leaves and/or roots, at PE, probably contributed to an enhanced Glc plant flux as supported by the high Glc levels that were detected in stems in response to defoliation. Furthermore, the defoliation-induced transcript accumulation of certain invertase genes in defoliated *A. cruentus* plants agreed with an increased expression of invertase genes in response to defoliation in *Lolium perenne* (Liu et al., 2015). In this grass species, a connection with regrowth was established between lowered Suc levels resulting from induced invertase activity and facilitated gibberellin biosynthesis through the up-regulation of Lp*GA2ox*, a gibberellin-activating enzyme gene.

Similar to maize, where energy deprivation conditions such as those imposed by prolonged darkness led to induction of most maize class II *TPS* genes examined (Henry et al., 2014), severe defoliation in *A. cruentus* produced an extensive up-regulation of these genes, particularly *AhTPS9* and *AhTPS11.* In concordance with their presumed function as sugar/Tre6P sensors able to ensure survival during stress conditions leading to C starvation (Arias et al., 2014; Delorge et al., 2014), these genes were also widely induced in leaves and roots of grain amaranths and *A. hybridus* in response to severe water-deficit stress (González-Rodríguez et al., 2019). Other studies have shown that class II TPS proteins have differential sensitivity to Suc levels in plants (Schluepmann and Paul, 2009), which was important in the context of modified defoliation tolerance observed at PE and FL. Likewise, the induction of *TPS11* and *TRE*, detected in stomatal guard cells of Suc-treated Arabidopsis (Bates et al., 2012), was interpreted as evidence of the proposed relationship between Tre, class II TPSs, carbohydrate metabolism, and stomatal movements *via* sugar sensing. The up-regulation of *AhTPS9* and *AhTPS11* by both water-deficit and defoliation stress also coincided with data showing that Suc-limiting conditions led to the induction of the *AtTPS8*–*AtTPS11* genes in Arabidopsis (Ramon et al., 2009; Baena-González and Hanson, 2017). Moreover, *TPS6, TPS8, TPS9, TPS10*, and *TPS11* were recognized as genes belonging to the sugar starvation network in *A. thaliana* (Arias et al., 2014). Furthermore, the overexpression of *OsTPS9* in rice significantly increased its tolerance toward cold and salinity stress (Zang et al., 2011). Several other experimental evidences support the regulatory role of these genes not only in stress conditions but also during development and in plant–microbe interactions. Thus, *PvTPS9* was proposed to be a crucial modulator of Tre metabolism in symbiotic nodules and whole common bean plants (Barraza et al., 2016); *OsTPP7* promoted early seed vigor by its regulation of Suc levels during germination under anoxic stress vigor (Kretzschmar et al., 2015); *MtTPS7* and *MtTPS10* were identified as early markers of the seed response to osmotic stress in *Medicago truncatula* (Macovei et al., 2019); class II *TPS* genes were associated with high-temperature stress resistance in *Moringa oleifera* (Lin et al., 2018); carbon starvation strongly induced the expression of *TPS7* and *TPS11* during early kiwi fruit development (Nardozza et al., 2020), and *TPS6, TPS7, and TPS11* were identified as yield-related traits in wheat (Lyra et al., 2020). In this context, the extensive neutral-to-negative effect that the defoliation stress had on the expression of the *AhTPS6, 7, 8*, and *9* genes in all organs examined, at FL, suggests that these genes might be essential to ensure an efficient recovery from defoliation in *A. cruentus*. Similarly, the highly contrasting class II *TPS* expression patterns detected in panicles, at PE and FL, suggests that they may also contribute to their opposite defoliation tolerance.

The widespread distribution of RFOs in higher plants obeys to their function as C stores and, also, as desiccation tolerance factors (Chaves and Oliveira, 2004; ElSayed et al., 2014). Previous studies revealed that RFOs probably contributed to water-deficit stress tolerance in quinoa genotypes (Bascuñán-Godoy et al., 2016), alfalfa (Kang et al., 2011), and grain amaranth (González-Rodríguez et al., 2019). RFOs accumulation in *A. thaliana* also resulted in enhanced resistance to drought, salinity, or cold (Taji et al., 2002; Panikulangara et al., 2004). Contrary to these reports, the contribution of RFOs to defoliation tolerance was not obvious, except for the peak accumulation of Raf in leaves of defoliated *A. cruentus* plants, at PE. Moreover, the extensive defoliation-related downregulation of RFO biosynthetic genes contrasted with other studies in which the expression of GolS genes correlated with abiotic stress tolerance in Arabidopsis (Taji et al., 2002; Nishizawa et al., 2008) and coffee (dos Santos et al., 2011, 2015).

## Conclusion

The present study identified several factors that were associated with the contrasting DT observed in *A. cruentus* plants defoliated at PE and FL, respectively. Among these were sharp differences in C allocation patterns, as represented by Suc levels and starch reserves in the plant. In addition to sucrolytic and amylolytic enzymes, regulation of C allocation patterns in response to severe defoliation appeared to be influenced by class II TPS genes, which are considered to actively participate in the regulation of C partition in plants during stress.

## Data Availability Statement

The original contributions presented in the study are included in the article/Supplementary Materials, further inquiries can be directed to the corresponding author/s.

## Author Contributions

JD-F, IC-H, and EV-O conceived, designed the experiments, and revised the manuscript. IC-H, EV-O, ES-M, and NM-G performed the experiments. JD-F, IC-H, EV-O, and DS analyzed the data. JD-F wrote the manuscript. IC-H, EV-O NM-G, and DS contributed to the reagents, materials, and analysis tools. All authors read and approved the final version of the manuscript.

## Conflict of Interest

The authors declare that the research was conducted in the absence of any commercial or financial relationships that could be construed as a potential conflict of interest.

## Footnotes

1 http://bioinfo.ut.ee/primer3/

2 http://www.premierbiosoft.com

3 http://www.idtdna.com/UNAFold

4 http://www.oligoevaluator.com

5 http://www.R-project.org

## Dedication

This article is dedicated to the memory of Axel Tiessen Favier. May he rest in peace.
