# Supplementary figures and images for: Highest Defoliation Tolerance in Amaranthus cruentus Plants at Panicle Development Is Associated With Sugar Starvation Responses
Source: Front Plant Sci. 2021 Jun 7;12:658977. doi: 10.3389/fpls.2021.658977 (PMC8215675; doi:10.3389/fpls.2021.658977)

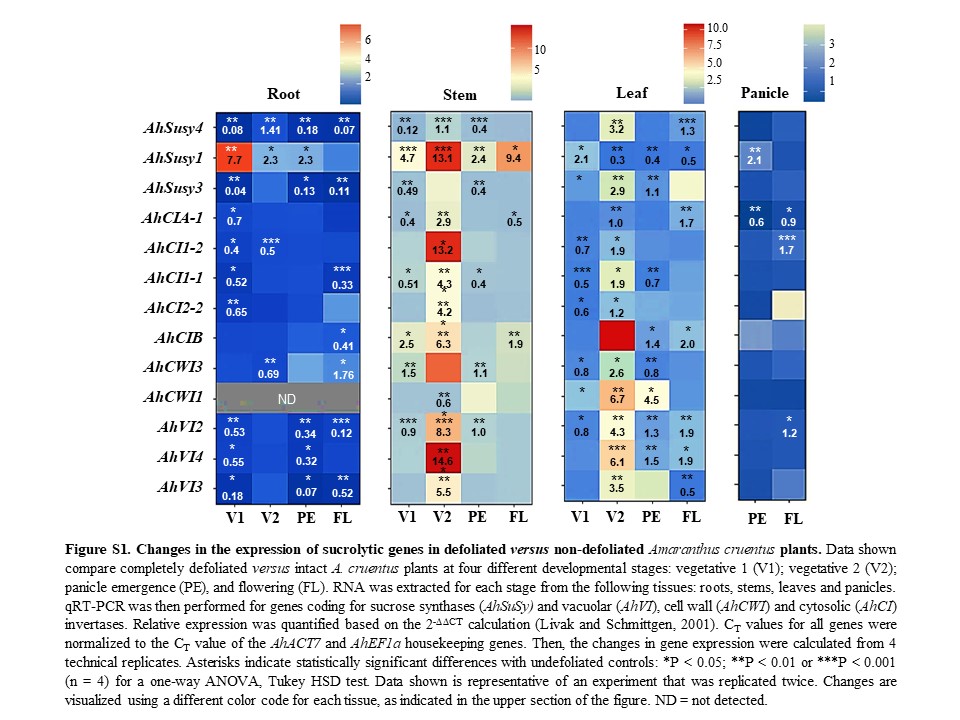

Supplement: Supplementary file 1 [file Image_1.jpg]

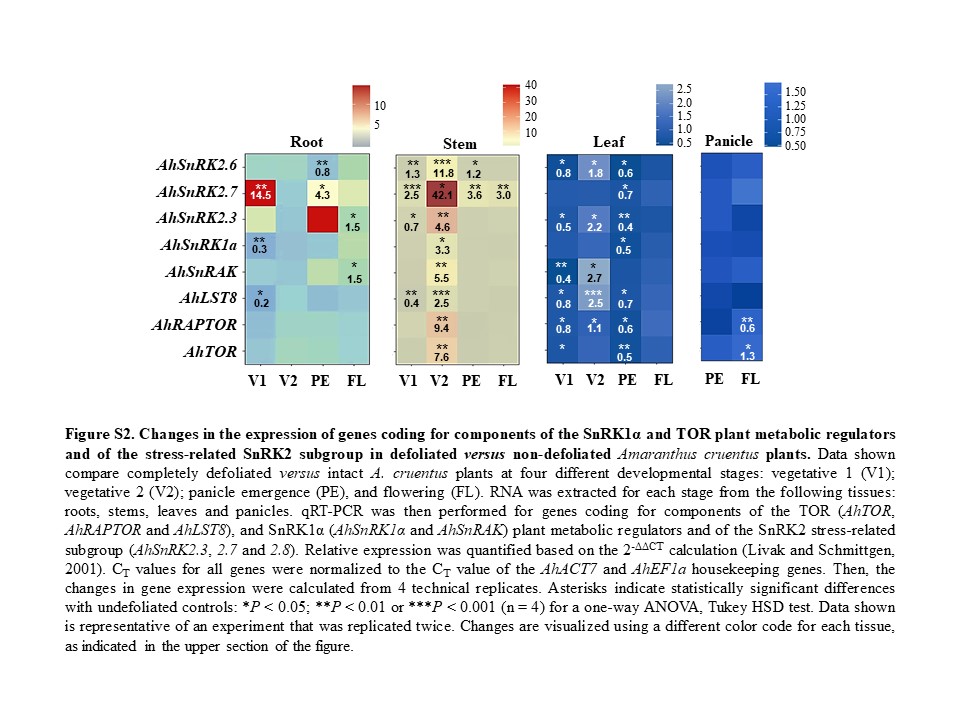

Supplement: Supplementary file 2 [file Image_2.jpg]

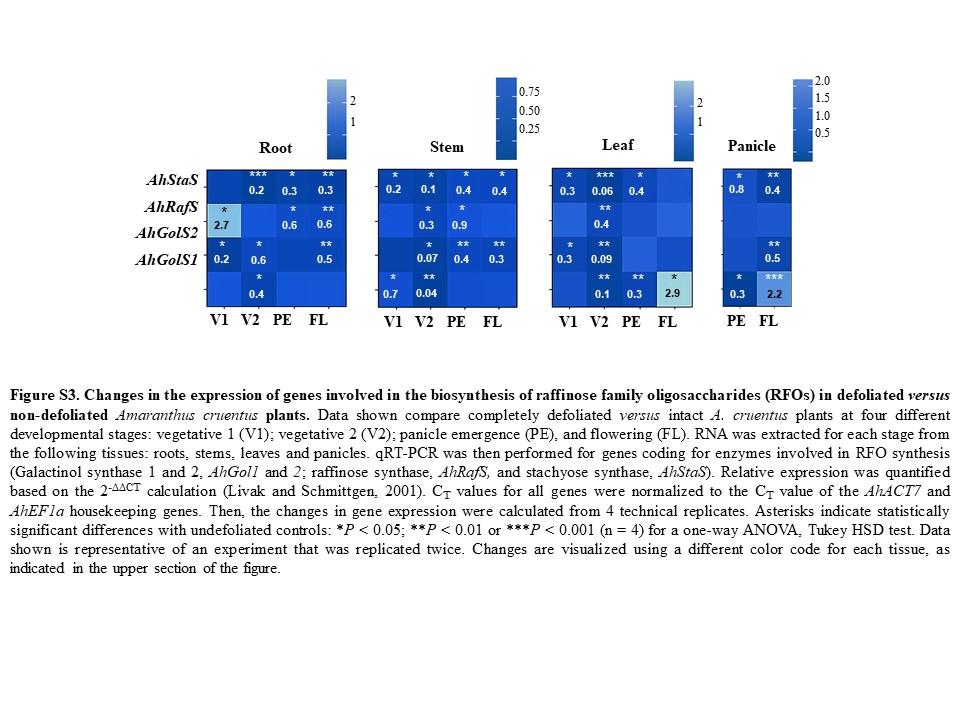

Supplement: Supplementary file 3 [file Image_3.jpg]

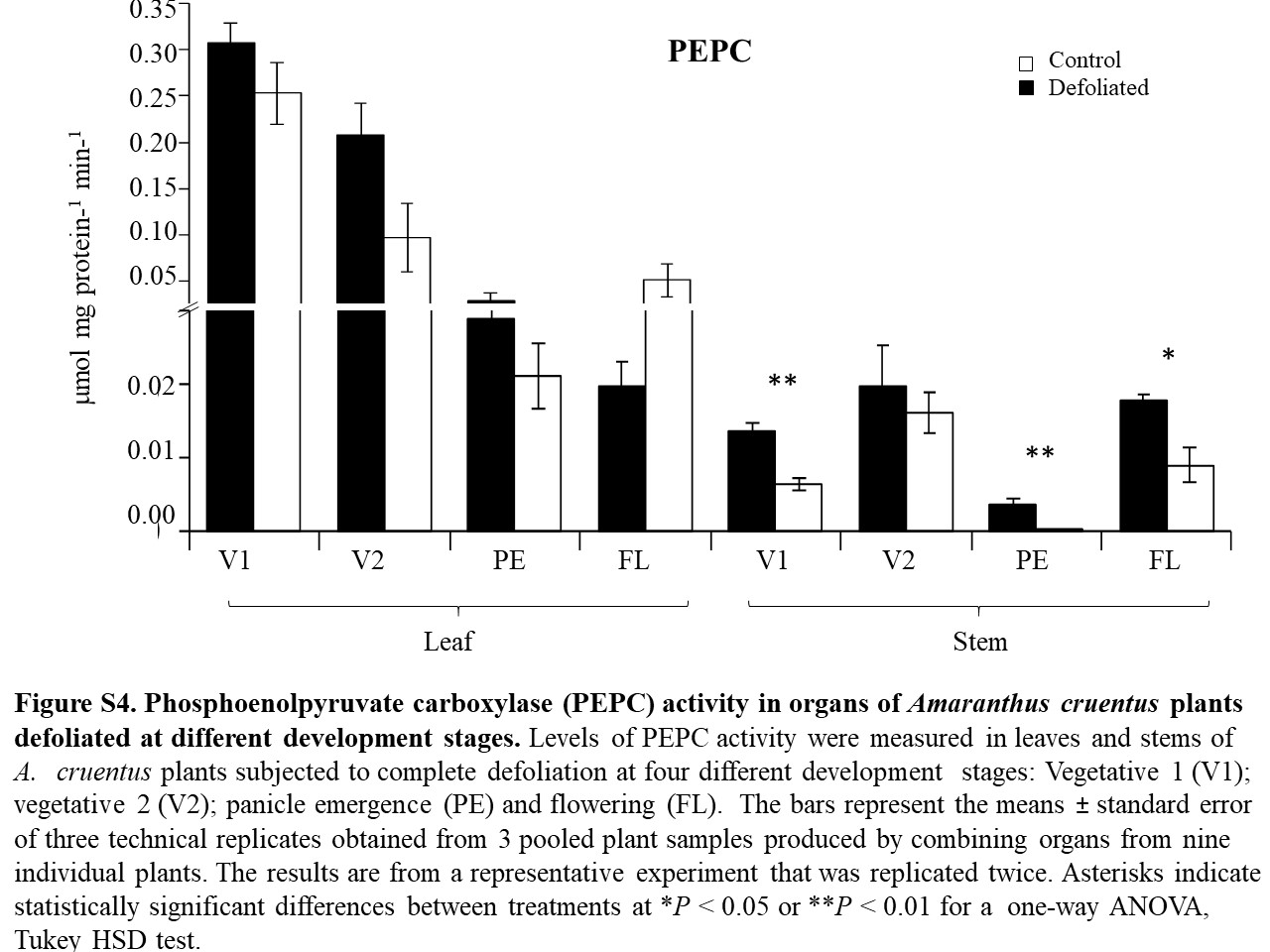

Supplement: Supplementary file 4 [file Image_4.jpg]
