# Supplementary material for: Highest Defoliation Tolerance in Amaranthus cruentus Plants at Panicle Development Is Associated With Sugar Starvation Responses
Source: Front Plant Sci. 2021 Jun 7;12:658977. doi: 10.3389/fpls.2021.658977 (PMC8215675; doi:10.3389/fpls.2021.658977)
Supplement: Supplementary file 5 [file Table_1.DOCX]

**Table S1**. Characteristics of the primers used for gene quantitation by qPCR.

| Gene | Sequence (5’🡪3’) | Tm (°C) | Length  (bp) | ΔG dimer  (kcal/mol) | ΔG stem-loop  (kcal/mol) | Amplificaction  efficiency (%) |
| --- | --- | --- | --- | --- | --- | --- |
| *AhVI2* | CAGGATCGGTTGTGCACTTGTATGTCA | 73.2 | 27 | - 7.0 | NF^1^ | 91.0 |
|  | ATCATCAGCAAGAACCAGAAGACCGA | 71.4 | 26 | N/E | NF |  |
| *AhVI4* | TCATCATATTCGTTATATCCCG | 60.1 | 22 | - 0.6 | - 0.6 | 99.7 |
|  | GATTCTGCTCATCTTGGTCC | 61.3 | 20 | - 0.5 | - 0.5 |  |
| *AhVI3* | ATACCCTGCCAACCTATCG | 61.3 | 19 | 0.5 | 0.5 | 106.5 |
|  | GATTACAATTCTCCATATTCCGT | 59.7 | 23 | - 1.1 | 0.0 |  |
| *AhCWI1* | TGGGGATAGAAGTATAATATAAT | 52.2 | 23 | - 0.5 | 0.5 | 104.1 |
|  | GCTGAAAATGATAGGCTGTA | 57.0 | 20 | N/E | NF |  |
| *AhCWI3* | TCAACTCAATCTCCCTATGTCA | 61.1 | 22 | N/E | NF | 93.6 |
|  | TGGGTGTGAAGTATGATTATGG | 61.1 | 22 | N/E | NF |  |
| *AhCI1* | ATATTGCTTCTTCATTGATGTTTG | 60.8 | 24 | - 0.9 | N/E | 81.3 |
|  | TAATTTTATCGAACAATGTCTTTTGAA | 62.4 | 27 | - 3.1 | - 0.7 |  |
| *AhCI1-2* | CTAGAGATATGTGTTTGTGCCCTGAC | 65.6 | 26 | - 1.6 | NF | 91.8 |
|  | AACATCTCCCCCATTTTCTTAGCT | 65.7 | 24 | - 3.0 | NF |  |
| *AhCI1* | ACGGATGAGGATTCTGAACT | 60.1 | 20 | - 0.6 | N/E | 92.9 |
|  | CAGCAGGTATCTTAGTTGATGTG | 60.3 | 23 | - 0.4 | - 0.4 |  |
| *AhCI2-2* | GCTAATGAAACCGCTGATTA | 59.4 | 20 | - 0.7 | - 0.7 | 95.1 |
|  | ACACAACCTAGACTTTGCCA | 59.8 | 20 | - 1.6 | - 0.7 |  |
| *AhCI-B* | ATTCTACATTAGCCGAACTGC | 59.8 | 21 | - 0.6 | - 0.6 | 99.0 |
|  | TCATACAACAACCATCAGCAC | 61.1 | 21 | N/E | NF |  |
| *AhSuSy3* | CCGTCGTGAGGCTCGTCGTTACC | 74.5 | 23 | - 1.0 | NF | 93.1 |
|  | CGCCATCGTACAATCAGTGCACATTC | 73.5 | 26 | - 6.9 | 0.0 |  |
| *AhSuSy1* | GAAATCCGTACCTCTGGCATC | 64.5 | 21 | - 2.0 | NF | 92.0 |
|  | CACTATACAAAACAAGCAGGAAAATA | 61.1 | 26 | - 0.3 | 0.5 |  |
| *AhSuSy4* | AAGCCATGTGACTCTCATACTG | 60.8 | 22 | - 2.3 | - 0.9 | 97.9 |
|  | CTAATTGCTATGAACCATACCG | 59.9 | 22 | - 1.3 | - 1.3 |  |
| *AhTPS1* | CTCCTTACAGTTGAGAAATCGC | 61.7 | 22 | - 1.0 | - 1.0 | 91.1 |
|  | TAACACTCTTGACCCAGATCG | 61.7 | 21 | - 2.0 | - 0.7 |  |
| *AhTPS5* | ATATAGGACATTGCCCGTGAG | 62.6 | 21 | - 0.5 | NF | 91.0 |
|  | CCAGCCCTAACATACGACTAC | 60.1 | 21 | N/E | NF |  |
| *AhTPS11* | AACATCCTTAGTATCTCGCCTG | 61.1 | 22 | N/E | NF | 98.0 |
|  | AGAACAACTCCTTCACTCTCG | 60.1 | 21 | - 0.7 | - 0.7 |  |
| *AhTPS6* | TTCAGCAACAGCATAATACGC | 62.7 | 21 | N/E | NF | 84.9 |
|  | GGAAACACAGAGGGAGACATAT | 60.8 | 22 | - 0.5 | NF |  |
| *AhTPS7* | AGGAAGCGATATTGAGGAGA | 60.4 | 20 | - 0.5 | NF | 90.4 |
|  | GCAATGGTGAAGTAAGCGAT | 61.7 | 20 | N/E | NF |  |
| *AhTPS8* | CACCATTAGTAGGTCCAACAG | 58.6 | 21 | - 1.5 | - 1.5 | 88.7 |
|  | CAAGTGATGATGAGCCAGG | 62.0 | 19 | N/E | NF |  |
| *AhTPS9* | TTCTTGCTCTGCCACTCAC | 61.9 | 19 | N/E | NF | 107.0 |
|  | ATTGTCTCGGCGTATAGGA | 59.7 | 19 | - 0.3 | NF |  |
| *AhTPS10* | TCCTTGAAAGACGGTCAGAAT | 62.6 | 21 | - 1.1 | - 0.9 | 83.3 |
|  | CTGCTTGTGTGCTTGAAGTTA | 60.7 | 21 | - 0.5 | - 0.5 |  |
| *AhTPPA* | TGTTCGTATTACTGCTTGGC | 60.3 | 20 | - 0.3 | NF | 90.8 |
|  | TGAGAGGGCTGACGGATA | 62.1 | 18 | N/E | NF |  |
| *AhTPPD* | GCAGATGGATGATTGACC | 58.9 | 18 | N/E | NF | 99.6 |
|  | TTCCAACTCCTCCTCCTTC | 60.8 | 19 | N/E | NF |  |
| *AhTPPI* | TTGGATTACGATGGCACTCT | 62.1 | 20 | N/E | NF | 103.5 |
|  | GGCACCTTCCACTCACTATT | 60.6 | 20 | N/E | NF |  |
| *AhTRE* | AACAACGGTAACGGTAGGAG | 60.5 | 20 | N/E | NF | 105.6 |
|  | GCAAGCACAGATCAATGC | 60.6 | 18 | - 2.0 | - 2.0 |  |
| *AhTOR* | GCACGCTCATTTAGAACCT | 59.5 | 19 | N/E | NF | 92.8 |
|  | CTACTCTTGGCAGCACTCAT | 59.7 | 20 | N/E | NF |  |
| *AhRAPTOR* | CATTGTATTCAGTTTGGCAT | 57.5 | 20 | N/E | NF | 99.4 |
|  | GGTGACATTATTGAGTTCCATA | 57.9 | 22 | 0.5 | 0.5 |  |
| *AhLST8* | TCATTTCGGTGTAGGGAAC | 60.0 | 19 | - 0.6 | - 0.6 | 94.5 |
|  | GGTCATCATCTCGGATCAA | 61.1 | 19 | - 2.0 | - 0.4 |  |
| *AhSnRAK* | CTCCTGTAGTGTCTCTCCGATA | 60.0 | 22 | N/E | NF | 101.9 |
|  | CCCGAATGTTGCTTAGGT | 60.1 | 18 | N/E | NF |  |
| *AhSnRK1α* | GGACCTCAGATGCTCTTCCTT | 60.0 | 21 | - 1.4 | - 1.4 | 102.2 |
|  | TGAAACACGACCATACATCAT | 60.3 | 21 | - 0.9 | - 0.9 |  |
| *AhSnRK2.6* | ATCCCAAAGTCTGAACAAGTA | 57.7 | 21 | N/E | NF | 109.8 |
|  | CGGCTCATTACAATCGTTT | 60.1 | 19 | 0.0 | NF |  |
| *AhSnRK2.7* | TCACAACAGAGGGACAATAAA | 59.6 | 21 | N/E | NF | 102.3 |
|  | GCTCTCTTCTTGGGTGGA | 60.6 | 18 | N/E | NF |  |
| *AhSnRK2.3* | GCCTTGACTCTCCTCGCC | 65.2 | 18 | N/E | NF | 106.6 |
|  | TGCTTCTCGGTATGACGG | 62.5 | 18 | N/E | NF |  |
| *AhGOLS1* | AGCCCAACTACACCCTTTATG | 61.7 | 21 | N/E | NF | 101.1 |
|  | TAATGGCACCAACAAACTTCG | 64.6 | 21 | N/E | NF |  |
| *AhGOLS2* | TCCTCCTACTCAAACTATCCAA | 59.3 | 22 | N/E | NF | 95.3 |
|  | AAAGGATCTAAGCAGCAGAAG | 59.3 | 21 | N/E | - 0.7 |  |
| *AhRAFS* | AAGCAGTTGAGTCACTATTCCA | 60.4 | 22 | N/E | - 0.8 | 95.5 |
|  | AGATGGTTCGTCTTGGCATAA | 63.3 | 21 | N/E | - 0.7 |  |
| *AhStaS* | CAATCCAAGGCAAATCAAACAC | 65.2 | 22 | N/E | NF | 90.5 |
|  | TCAAATGAGGCACCAAAGAAG | 64.0 | 21 | N/E | NF |  |
| *AhACT7* | CGTGACCTGACTGATTACCTTA | 60.6 | 22 | N/E | NF | 101.2 |
|  | GCTCGTAGTTCTTCTCAATGGC | 64.0 | 22 | N/E | NF |  |
| *AhEF1α* | GCCAAATATCTAAGAAACAAATGC | 61.5 | 24 | - 0.7 | NF | 104.9 |
|  | TAGCACAACCACATGATATTTCTT | 61.2 | 24 | - 2.3 | NF |  |

**^1^NF = Not Found.**
